# Early clinical outcomes and quality of life assessment after HeartMate 3 implantation: A single-centre descriptive study

**DOI:** 10.62838/jccm-2026-0015

**Published:** 2026-04-30

**Authors:** Samuel Michelini, Marius Mihai Harpa, Dan Nistor, David-Emanuel Anitei, Alexandru Stan, Paul Calburean, Emil Marian Arbanasi, Dragos-Florin Baba, Sabrina Mancino, Horatiu Suciu

**Affiliations:** George Emil Palade University of Medicine Pharmacy Science and Technology of Targu Mures, Targu Mures, Romania; Department of Cardiovascular Surgery, Emergency Institute for Cardiovascular Diseases and Transplantation, Targu Mures, Romania; Doctoral School, George Emil Palade University of Medicine, Pharmacy, Science and, Technology of Targu Mures, Targu Mures, Romania; Department of Biostatistics and Medical Informatics, George Emil Palade University of Medicine, Pharmacy, Science and Technology of Targu Mures, Targu Mures, Romania; Department of Vascular Surgery, George Emil Palade University of Medicine, Pharmacy, Science and Technology of Targu Mures, Targu Mures, Romania; Department of Cell and Molecular Biology, George Emil Palade University of Medicine, Pharmacy, Science and Technology of Targu Mures, Targu Mures, Romania; Department of Surgery IV, George Emil Palade University of Medicine, Pharmacy, Science and Technology of Targu Mures, Targu Mures, Romania

**Keywords:** advanced heart failure, HeartMate 3, perioperative complications, quality of life, psychosocial outcomes

## Abstract

**Background:**

Advanced heart failure remains a leading cause of morbidity and mortality worldwide, with limited access to transplantation, particularly in Eastern Europe. Left ventricular assist device therapy offers improved survival and quality of life for end-stage disease. HeartMate 3, a modern form of mechanical circulatory support, is used as bridge-to-transplant or destination therapy. Despite increasing global experience with the HeartMate 3, clinical data from Romanian centers remain scarce. This study aimed to assess early clinical outcomes, postoperative complications, and quality of life after HeartMate 3 implantation at a single cardiovascular surgery center in Romania.

**Materials and methods:**

We conducted a retrospective observational cohort study including 13 patients with Advanced heart failure who underwent HeartMate 3 implantation between September 2023 and March 2025. Preoperative variables (demographics, comorbidities, INTERMACS profile, EuroSCORE II, echocardiographic findings) and postoperative outcomes were analyzed. Quality of life was assessed in 10 surviving patients using a 16-item structured telephone questionnaire addressing physical function, autonomy, social reintegration, and psychological well-being.

**Results:**

The cohort was predominantly male (84.6%); mean age 47.2 ± 12.3 years. Implant strategy: bridge-to-transplant 76.9%, destination therapy 23.1%. Early mortality was 23.1% (n = 3), occurring primarily in patients with EuroSCORE II >8% and INTERMACS I–II. The most frequent postoperative complications were significant perioperative bleeding (61.5%) and right ventricular failure (23.1%). Among survivors, all reported improved mobility, greater independence in activities of daily living, and better social reintegration; 7/10 rated overall quality of life as good or excellent. Psychological distress was frequent early after surgery but showed progressive improvement over time.

**Conclusions:**

HeartMate 3 implantation resulted in favorable early clinical outcomes and significant improvements in quality of life, aligning with international data. Optimizing outcomes with left ventricular assist device therapy relies on timely referral, rigorous patient selection, and comprehensive postoperative management, including psychological and social support.

## Introduction

Heart failure (HF) is a complex clinical syndrome associated with impaired quality of life, frequent hospitalizations, and high morbidity, with a reported 1-year mortality of 25–50% [1,2,3,4]. Being a progressive disease, approximately 10% of the overall HF population suffers from end-stage disease, defined as advanced heart failure (ADHF), with persistent and/or progressive HF signs and symptoms despite optimal medical therapy (OMT), including cardiac resynchronization therapy or percutaneous mitral valve repair [5, 6].

Although over the last 50 years heart transplantation (HT) has been the gold standard for the treatment of ADHF [7], it remains a limited option for a highly selected patient population due to organ shortages, with demand far exceeding availability worldwide, particularly in Eastern European countries like Romania, where donor scarcity is even more pronounced.

Left ventricular assist devices (LVADs), on the other hand, represent a more readily accessible, life-saving therapy for various categories of ADHF patients. Over the last few years, LVAD therapy has experienced exponential development, with improved survival and quality of life, device reliability, and reduced complications. Patient selection, however, remains critical, as successful therapy depends on strategic timing of implantation and an accurate assessment of the risk–benefit ratio. The INTERMACS (Interagency Registry for Mechanically Assisted Circulatory Support) classification is routinely applied to categorize patients by clinical stability and urgency, thereby optimizing the choice between medical and mechanical support [8]. Beyond clinical criteria, psychosocial evaluation is equally important. Candidates must be motivated and able to adhere to therapy, as poor adherence to previous therapies, psychiatric illness, or cognitive impairment predict poor outcomes on LVAD [9, 10].

Complications and adverse events related to mechanical circulatory support constitute the main paradox in this field. Post-implant right ventricular failure (RVF) is one of the most feared complications associated with LVAD therapy and may severely impair patient outcomes. Furthermore, hemocompatibility-related adverse events (HRAEs), a spectrum including both increased bleeding risk and hypercoagulability remain clinically significant[11]. Infectious complications, particularly driveline infections, continue to be the Achilles' heel of LVAD therapy, with the potential to progress from local involvement to systemic sepsis.

The HeartMate 3^®^ LVAD (Abbott Laboratories, Chicago, IL, USA) represents the latest generation of mechanical circulatory support devices. Its fully magnetically levitated centrifugal pump has been associated with excellent outcomes in terms of both efficiency and safety, making it the global standard of care, emblematic of the exciting engineering potential and ongoing innovation present in HM3 and that define LVAD field [11].

Given the ongoing clinical and psychosocial challenges of LVAD therapy, the present study aims to provide an early clinical assessment of patients who have undergone HeartMate 3^®^ (HM3) implantation at the Emergency Institute for Cardiovascular Diseases and Transplantation in Târgu Mureș, Romania. Specifically, the study evaluates patient selection and risk stratification using EuroSCORE II, early postoperative complications and outcomes, and changes in quality of life (QoL) before and after LVAD implantation.

## Materials and methods

This was a retrospective observational cohort study that included 13 adult patients with advanced heart failure who underwent implantation of the HM3 LVAD at the Emergency Institute for Cardiovascular Diseases and Transplantation between September 2023 and March 2025.

Patient evaluation included demographic data, clinical classifications, comorbidities, preoperative echocardiographic parameters, EuroSCORE II calculation, postoperative outcomes, and quality of life assessment using a structured questionnaire. Ethical approval was granted by the institutional ethics committee (Reference number: 2884/2025). Data were collected from the operating room registries (2023–2025), the institutional database and echocardiography reports.

Given the descriptive nature of the study and the small sample size, no formal statistical comparisons were performed. Continuous variables are reported as mean ± standard deviation or median (range), as appropriate, while categorical variables are presented as counts and percentages. Analyses were performed using Microsoft Excel (Microsoft Corporation, Redmond, WA, USA).

Quality of life was assessed in 10 surviving patients using a 16-item questionnaire, an exploratory, non-validated instrument specifically designed for this study, to capture patient-reported functional, psychosocial, and device-related experiences relevant to early LVAD support (See Supplementary material). Interviews were conducted by telephone, with patients contacted in various regions of Romania where they currently reside.

Inclusion criteria consisted of all adult patients (≥18 years) who underwent LVAD implantation during the study period.

## Results

### Demographics

A total of 13 patients underwent HM3 implantation during the study period. The cohort was predominantly male (84.6%, *n* = 11), with a mean age of 47.2 ± 12.3 years (range: 18–69 years). The most frequent age group was 50–59 years (38.5%, *n* = 5). Postoperative length of stay was variable, with most patients (38.5%, *n* = 5) discharged within 20–30 days; one patient required prolonged hospitalization (>60 days). As of June 2025, four patients had survived on HM3 support for more than one year, another four for 6–12 months, and one for 3–6 months. The remaining three patients died between 17- and 45-days post-implantation.

### Functional Classifications

At the time of implantation, the majority of patients (*n* = 9) were classified as New York Heart Association (NYHA) Class IV, with the remainder (30.8%) in Class III. Patient distribution according to the Interagency Registry for Mechanically Assisted Circulatory Support (INTERMACS) classification was heterogeneous. One patient each was categorized in INTERMACS profiles I, II, and III, indicating critical preoperative status; two patients were in profile IV; and the majority (*n* = 8, 61.5%) were in profile V. In terms of therapeutic indication, patients were categorized based on eligibility for HT. Most patients (*n* = 10, 76.9%) received HM3 as bridge-to-transplant (BTT), while the remaining (*n* = 3, 23.1%) were implanted as destination therapy (DT).

### Comorbidities

Most patients presented with significant comorbidities in association with severe cardiac dysfunction. Obesity (Class I) and type 2 diabetes mellitus (DM) were each identified in 30.8% (*n* = 4) of the cohort. Renal impairment was observed in a subset of patients: two were classified as having stage G3 chronic kidney disease (CKD), one as stage G2, and one as stage G4, according to the Kidney Disease: Improving Global Outcomes (KDIGO) classification. Hepatic dysfunction was less frequent, with two cases of hepatocytolysis syndrome and one case of toxic hepatopathy. Pulmonary comorbidities were documented in three patients, two with chronic obstructive pulmonary disease (COPD) and one with bronchial asthma. A substantial proportion of patients also had a significant previous cardiovascular history relevant to the LVAD candidacy evaluation. These included myocardial infarction (*n* = 3), ischemic stroke (*n* = 3), cardiac arrest (*n* = 3), and recurrent pleural effusion (*n* = 3).

### Preoperative cardiac evaluation

All patients included in the study were diagnosed with dilated cardiomyopathy (DCM). Regarding DCM etiology, the most frequent was non-ischemic origin (*n* = 6, 46.2%), followed by ischemic (*n* = 3, 23.1%), idiopathic (*n* = 2, 15.4%), myocarditis-related (*n* = 1), and familial DCM (*n* = 1), the latter confirmed through genetic counseling. Left ventricular end-diastolic diameter (LVEDd) was severely increased in 11 patients (defined as >65 mm in men, >59 mm in women), while two patients exhibited moderate dilation. All patients had severely reduced left ventricular ejection fraction (LVEF <30%), with three cases presenting LVEF <20%. Pulmonary hypertension (PHTN), estimated by echocardiographic calculation of pulmonary artery systolic pressure (PAPs), was common: severe in 23.1% (*n* = 3), moderate in 46.2% (*n* = 6), mild in 7.7% (*n* = 1), and absent in only one patient.

PHTN severity was classified by Doppler echocardiography as mild (30–39 mmHg), moderate (40–49 mmHg), and severe (≥50 mmHg). Valvular dysfunction was identified in nearly all patients (*n* = 12), predominantly involving the atrioventricular valves. Mitral regurgitation was present in *n* = 12 patients (*n =*6 severe, *n =* 4 moderate), tricuspid regurgitation in *n =* 11 patients (*n =* 4 severe, *n =* 5 moderate), and aortic regurgitation in n = 6 patients (n = 5 mild, n = 1 moderate).

### EUROSCORE II risk stratification

The European System for Cardiac Operative Risk Evaluation II (EuroSCORE II) was applied to estimate the probability of operative and in-hospital mortality in patients undergoing HM3 implantation. This model incorporates a wide range of preoperative variables, including patient-related factors (age, sex, renal dysfunction based on creatinine clearance, extracardiac arteriopathy, impaired mobility, previous cardiac surgery, chronic pulmonary disease, active endocarditis, and critical preoperative status), cardiac-related factors (NYHA functional class, Canadian Cardiovascular Society class 4 angina, left ventricular systolic function, recent myocardial infarction within 90 days, and severity of pulmonary hypertension), as well as procedure-related parameters (urgency of surgery, procedural complexity, and whether thoracic aorta surgery was performed). Based on calculated EuroSCORE II values, the majority of patients (n = 7) were classified as intermediate risk (predicted mortality 4–8%), while four patients were categorized as high risk (>8%) and two as low risk (<4%).

### Postoperative outcomes

In the postoperative period, the majority of patients (*n* = 7) demonstrated a favorable clinical course from the outset. Three individuals experienced early postoperative complications that initially delayed recovery, though their outcomes were ultimately favorable. In contrast, three patients had an unfavorable evolution, culminating in death. Among early complications, RVF remained the most feared adverse event, occurring in three patients, as per INTERMACS definition. The most frequently observed complication was perioperative bleeding, occurring in the form of hematomas or serous-bloody fluid collections, specifically pleural effusion or hemothorax (*n* = 6) and pericardial effusion or hemopericardium (*n* = 2). Other complications included pneumonia (23.1%, *n* = 3) and RVF (23.1%, *n* = 3). Additional isolated events were recorded: ischemic stroke (*n* = 1), mesenteric thrombosis (*n* = 1), gastrointestinal bleeding (*n* = 1), coagulopathy with thrombocytopenia (*n* = 1), postoperative anemia (*n* = 2), acute kidney injury (*n* = 1), and postoperative delirium (*n* = 1).

### Assessment of Quality of Life (QoL)

The quality of life of the *n* = 10 surviving patients was evaluated through a structured telephone questionnaire designed specifically for LVAD recipients. The instrument assessed pre- and post-implantation status, independence in activities of daily living, psychological well-being, late complications, device management, patient concerns, and perceived benefits of the therapy. At the time of the survey, six patients lived in urban areas and four in rural regions; most (n = 8) had family caregivers. Before LVAD implantation, all patients rated their quality of life as very poor and described being completely dependent on caregivers. Following implantation, all participants reported substantial improvements. Three rated their quality of life as excellent, four as good, and three as fair. All patients regained the ability to walk independently and climb stairs. Six were able to shop for groceries without assistance, and seven resumed regular social interactions. However, three remained socially limited to interactions with close family members. Psychological adaptation during the early postoperative period was challenging for many. Six patients experienced symptoms of depression or anxiety, particularly within the first 4–8 weeks post-implantation. Three reported transient suicidal ideations during this phase, with one suffering an acute episode of suicidal behavior. Sleep disturbances were also common in the early postoperative period, with seven patients reporting insomnia, primarily due to difficulties adjusting to the driveline, batteries, or the pump noise. Over time, these symptoms improved either spontaneously or with targeted therapy. Notably, only two patients received structured psychological support, despite current guideline recommendations emphasizing its importance. Device-related complications were relatively common but manageable. Five patients reported local driveline infections or trauma, most commonly resulting from minor domestic incidents. Importantly, no patients experienced stroke, transient ischemic attacks, or gastrointestinal bleeding, although two reported sporadic episodes of epistaxis. Technical issues were infrequent. Three patients reported difficulties interpreting alarm signals, but no critical malfunctions occurred. Regarding confidence in device management, five patients reported feeling very confident, four were somewhat confident, and one reported low confidence. The daily management of the device, including driveline care and battery maintenance, required approximately 30 minutes. Four patients admitted to occasional anxiety related to battery life, particularly during power outages, though no adverse events were reported. None of the patients encountered difficulties in charging batteries or managing the device connections. Two patients required caregiver support for dressing and bathing, two managed these tasks independently but with difficulty, and six reported no limitations. Regarding mobility, most patients were able to leave their homes and travel; however, four preferred to remain close to home due to caregiver dependence.

Physical activity (PA) levels were encouraging: nine patients engaged in regular exercise (*n* = 8, >5 days/week; *n* = 1, 3–4 days/week), primarily walking and gym training. Six exercised for more than 60 minutes per day, two for 30–60 minutes, and one for 15–30 minutes. Only one patient reported a sedentary lifestyle. A minority of patients acknowledged experiencing occasional social isolation, but none described it as frequent or severe. All participants affirmed that the device had restored dignity and significantly improved their lives (Table 1).

**Table 1.** Key outcomes (demographics, baseline clinical status, complications, early outcomes, quality of life outcomes). NYHA-New York heart Association; INTERMACS-Interagency Registry for Mechanically Assisted Circulatory Support; COPD-Chronic Obstructive Pulmonary Disease; QoL-Quality of Life.

**Variable**	**Value**
Demographics	
Age, years (mean ± SD)	47.2 ± 12.3
Male sex, n (%)	11 (84.6)
Implant strategy – Bridge-to-transplant, n (%)	10 (76.9)
Implant strategy – Destination therapy, n (%)	3 (23.1)

Baseline clinical status	
NYHA class IV, n (%)	9 (69.2)
INTERMACS I–II, n (%)	2 (15.4)
INTERMACS III–IV, n (%)	3 (23.1)
INTERMACS V, n (%)	8 (61.5)
EuroSCORE II low risk (<4%), n (%)	2 (15.4)
EuroSCORE II intermediate risk (4–8%), n (%)	7 (53.8)
EuroSCORE II high risk (>8%), n (%)	4 (30.8)

Postoperative complications	
Perioperative bleeding, n (%)	8 (61.5)
Right ventricular failure, n (%)	3 (23.1)
Pneumonia, n (%)	3 (23.1)
Ischemic stroke, n (%)	1 (7.7)
Gastrointestinal bleeding, n (%)	1 (7.7)

Early outcomes	
Early mortality (≤45 days), n (%)	3 (23.1)
Survivors at follow-up, n (%)	10 (76.9)

Quality of life outcomes	
Reported overall QoL improvement, n	10
QoL rated good or excellent, n	7
Independent ambulation regained, n	10
Resumed regular social interactions, n	7
Early postoperative psychological distress, n	6
Regular physical activity (>3 days/week), n	9
Sleep disturbances early post-implant, n	7
Transient suicidal ideation post-implant, n	3
Reported confidence in device management, n	9
Local driveline infection/trauma, n	5

## Discussions

The results demonstrate both the challenges and the benefits of LVAD therapy in ADHF, while also reflecting the global trends in device utilization and patient outcomes. Over the past decade, LVAD therapy has undergone rapid expansion, largely due to an exponential technological improvement and better patient selection. Between 2014 and 2024, the number of LVAD implantations increased tenfold, with 29,634 continuous-flow devices now registered in the INTERMACS registry [12].

The present findings align with international experience, confirming the safety and clinical benefits of HM3 therapy. Our patient population showed an 85% male predominance, consistent with global registries reporting that over 80% of LVAD and transplant recipients are men [13]. This is likely due to the higher prevalence of HF with reduced ejection fraction in men, while women are more often affected by HF with preserved ejection fraction, which is less frequently treated with LVADs [14]. Regarding indications for implantation, most patients underwent LVAD surgery as a BTT. In Romania, the maximum eligible age for transplantation is 60 years, which is lower than in many European centres where the limit is 65 years. As a result, older patients or those with significant comorbidities such as CKD, COPD, diabetes or prior CABG were listed as DT candidates. International literature confirms that DT patients are generally older and present with more frequent renal impairment and prior revascularization procedures.

Nevertheless, the MOMENTUM 3 trial demonstrated that HM3 provides similar two-year outcomes in both BTT and DT patients [6, 15]. These findings, coupled with the fact that some patients initially implanted for DT may achieve sufficient clinical improvements to become transplant eligible, and that patients originally implanted for BTT may develop contraindications to HT such as disabling stroke [15], imply that distinguishing between DT and BTT indications may not be as significant nowadays[16].

Careful assessment of comorbidities remains crucial. In this series, four patients had CKD of varying severity. While end-stage renal disease requiring dialysis remains an absolute contraindication, moderate dysfunction is not [6, 17], though it can worsen prognosis. Similarly, three patients presented with mild hepatic dysfunction, which does not preclude implantation, whereas severe or irreversible dysfunctions are consistently linked with poor outcomes [10], linked to the high bleeding risk and protein synthesis deficit. DM (Type 2) was present in 31% of patients, aligning with published data [18], and is an important predictor of infection and late mortality in LVAD patients [19]. Obesity (Class I), found in four patients, is increasingly recognized as not prohibitive, indeed, the so-called “obesity paradox” suggests some survival benefit in obese patients with HF, therefore not representing a contraindication for LVAD therapy [20].

By consultation of echocardiographic data and assessment of the cardiac history, the etiology of HF was established, all patients in this study were diagnosed with DCM, predominantly of non-ischemic etiology. This is an important finding, as restrictive or hypertrophic cardiomyopathies, such as amyloidosis or radiation-induced cardiomyopathy, are associated with worse outcomes compared to dilated or ischemic forms [21]. Most of our patients had severely dilated ventricles, with LVEDd values exceeding the prognostic cut-off of 59 mm [6], which is associated with better device performance and post-implant prognosis [22].

As predictable, in the presence of DCM, valvular disease was almost universal. Literature shows that severe mitral regurgitation often improves after implantation because of ventricular unloading, hence it does not represent a contraindication [23]. Tricuspid regurgitation, on the other hand, is closely associated with post-implant RV failure, hence any moderate or severe regurgitation should be assessed, and repair or replacement should be performed [24-25]. The dilemma remains whether leaving the patient with some degree of insufficiency could help reducing the overload of the RV due to the high PAPs, for this reason repair or replacement was not performed in any of the patients in this study (n=3 severe; n=5 moderate), but evaluation of the early postoperative outcomes in the patients presenting with severe TR revealed the onset of RVF and consequent catastrophic sequalae. In case of aortic regurgitation, biological valve replacement or repair is needed in the presence of more than mild regurgitation, hence aortic valvuloplasty by central suture was performed during LVAD implantation to the only subject presenting with moderate insufficiency.

Postoperative survival and predicted surgical risk were calculated using the EuroSCORE online calculator[26].

EuroSCORE II appeared useful and consistent with the results, as three of four patients classified as high-risk died within 45 days of implantation because of the consequences of a very poor preoperative status associated with severe INTERMACS profiles (I and II) and severe comorbidities such as G4 CKD.

Other studies have confirmed that EuroSCORE II may be useful to predict survival in LVAD patients and it proved to be superior to INTERMACS after 1 year [27]. Timing of implantation in the “golden window” is another key determinant: historically, patients in INTERMACS profiles II–III tend to benefit most [6], while those in profile I often have prohibitive risk due to irreversible organ dysfunction and a higher risk of adverse events post-implant. In this study, patients in profile IV and V achieved the most favourable early outcomes, suggesting that the new technologies present in HM3, the reduced rate of postoperative complications and a more conscious candidate selection process allow “early” LVAD implantation [28, 29].

The most frequent perioperative complications were haemothorax or hemopericardium, observed in nearly half of patients, requiring in all cases surgical or non-surgical evacuation. This finding is consistent with other reports identifying bleeding as one of the most common early complications [30]. Right ventricular failure emerged as the leading cause of mortality; An already severe hemodynamic instability was aggravated in all cases by other dysfunctions such as coagulopathies, thrombocytopenia, bleeding, thromboses, stroke or pneumonia with the subsequent development of multiorgan failure terminated in all the three cases in the death of the patient. Post-implant RVF was defined according to established INTERMACS criteria, as the presence of clinical and hemodynamic signs of RV dysfunction, requiring prolonged inotropic or vasopressor support, mechanical right-sided circulatory support, or resulting in end-organ dysfunction. Clinical features included persistent hypotension, elevated central venous pressure, low cardiac output despite adequate LVAD function, a picture of refractory hemodynamic instability ultimately leading to multiorgan failure. Literature emphasizes that RVF typically develops within two weeks of implantation and is difficult to predict or treat, despite OMT [11]. Although temporary right-sided mechanical support such as Extracorporeal Membrane Oxygenation (ECMO) or percutaneous RV assist devices can improve prognosis in high-risk patients [31,32,33], our single ECMO-supported patient did not survive, reflecting the high mortality associated with severe RVF.

Perhaps the most striking finding was the radical improvement in QoL among survivors. In LVAD patients, caregiver support is essential; about 80% relied on family members for daily activities, therapy adherence, and driveline care. The absence of such support is considered a major contraindication to implantation.

All patients reported very poor quality of life prior to implantation; following LVAD therapy, most described their quality of life as “good” or “excellent”, with marked improvements in mobility, independence, and social reintegration. This study showed that increased confidence with the device over time translated into greater freedom, including the ability to travel long distances, even by plane, with higher QoL ratings consistently reported at longer durations after implantation.

The postoperative course was not without challenges. Psychological distress was common, of the three patients reporting transient suicidal thoughts, in two cases the ideation was spontaneously disclosed during interviews and described as passive, transient thoughts without intent or planning, occurring in the context of psychological burden of device dependency, extended hospitalization, and the demands of daily self-care. In one case, however, the patient experienced an acute episode of suicidal behavior during inpatient recovery in the early postoperative period. Management occurred directly within the hospital setting, involving immediate staff intervention and appropriate clinical oversight according to institutional safety protocols. Whenever significant psychological distress was disclosed or observed, patients were advised to seek psychological or psychiatric support through existing institutional or community-based referral pathways. No further suicidal behavior was reported thereafter, nevertheless, the identification of both transient suicidal ideation and one episode of inpatient suicidal behavior highlights the psychological vulnerability of some patients during the early postoperative phase of LVAD implantation and highlights the importance of systematic psychological monitoring and accessible referral pathways during this period, as suggested by many guidelines; At present only two patients included in this study received it.

Driveline infections, inflammation or trauma occurred in half of the patients. These were successfully treated with oral antibiotics or topical therapy, and none progressed to systemic infection. Being exposed to the external environment and managed by patients with routine dressing changes[11], it constitutes an easy portal of entry. The most common pathogens involved in LVAD infections are the ones from the skin flora (Staphylococcus epidermidis and aureus) and enteric gram-negative rods such as Pseudomonas and Klebsiella, with polymicrobial infections being identified in over half of cases[34]. In the ADHF population, the main risk factors for infection include older age, diabetes, renal dysfunction, obesity, prolonged duration of LVAD therapy and history of trauma to the driveline site[35].

Although presenting with much lower incidence rates than in the past, bleeding, pump thrombosis and stroke continue to be important risks related to LVAD therapy[36]. No cases were recorded in our study, confirming that experience with HM3 LVAD suggests a marked reduction in stroke rates, compared to both HeartMate 2 and HVAD with an incidence that has been recently registered around 14% at five years[12]. Thrombotic complications rates have dropped so significantly that anticoagulation therapy alone is now the mainstay of treatment, in comparison to the warfarin and aspirin regimen previously adopted. Patients in our institution are treated only with vitamin K antagonists in the absence of comorbidities requiring aspirin, as confirmed in the recent ARIES-HM3 trial, the total exclusion of aspirin was demonstrated to be safe if Vitamin K antagonists are used for an INR target of 2.0–3.0, with a net reduction of bleeding events[37].

All participants highlighted increased independence, renewed participation in family and social activities, and the ability to resume physical exercise for good physical recovery and mental health.

Although the initial postoperative period was universally described as the most difficult phase, both physically and emotionally, patients gradually adapted to the device, anxiety subsided, and the advantages of regained autonomy became increasingly evident.

In our experience, HeartMate 3 therapy proved feasible and effective, with outcomes comparable to international reports. The early mortality in high-risk patients illustrates the need for earlier referral, while the marked improvements in QoL among survivors highlight the transformative potential of this therapy. Broader implementation of structured psychological support, rigorous driveline care protocols, and timely recognition of right ventricular dysfunction may further improve outcomes.

### Limitations of the study

This study has several important limitations. First, the small sample size (n=13) limits statistical power and precludes formal inferential analysis; results should be considered exploratory. Second, it was conducted at a single centre, limiting generalizability to broader populations. Third, validated quality-of-life instruments such as the Kansas City Cardiomyopathy Questionnaire (KCCQ) or EQ-5D were not used due to the retrospective, telephone-based assessment, limited sample size, and variable follow-up. The questionnaire was designed to focus on LVAD-specific aspects of daily living, psychosocial adaptation, and device management. Therefore, quality-of-life findings should be interpreted descriptively. Additionally, retrospective telephone interviews may introduce recall bias, and assessment limited to surviving patients could result in survivor bias. Fourth, the follow-up duration was heterogeneous, with some patients having less than 6 months of post-implant observation.

## Conclusion

Despite the challenges, this single-center study confirms that HeartMate 3^®^ offers favorable early outcomes with a profound and sustained improvement in quality of life for patients who would otherwise have no viable treatment options.

Early mortality was associated with high EuroSCORE II and critical INTERMACS profiles, emphasizing the importance of timely referral and appropriate patient selection. RVF and bleeding were the most frequent early complications, while late adverse events were limited, with no stroke or major bleeding reported.

HM3 demonstrated high device reliability and excellent patient adaptation. Optimizing outcomes requires early implantation in stable candidates (INTERMACS III–V), accurate preoperative risk stratification, and structured postoperative support, including psychological care.
